# Comprehensive analysis of differentially expressed profiles of Alzheimer’s disease associated circular RNAs in an Alzheimer’s disease mouse model

**DOI:** 10.18632/aging.101387

**Published:** 2018-02-15

**Authors:** Jin-Lan Huang, Mei-Chun Qin, Yan Zhou, Zhe-Hao Xu, Si-man Yang, Fan Zhang, Jing Zhong, Ming-Kun Liang, Ben Chen, Wen-Yan Zhang, Deng-Pan Wu, Zhen-Guo Zhong

**Affiliations:** 1 Department of Pharmacology, Pharmacy School, Xuzhou Medical University, Xuzhou, Jiangsu 221004, China; 2 Scientific research center of traditional Chinese medicine, Guangxi University of Chinese Medicine, Nanning, Guangxi 530200, China; 3 Jiangsu Key Laboratory of New Drug Research and Clinical Pharmacy, Pharmacy School, Xuzhou Medical University, Xuzhou, Jiangsu 221004, China; 4 Ruikang Hospital, Guangxi University of Chinese Medicine, Nanning, Guangxi 530200, China; * Equal contribution

**Keywords:** Alzheimer’s disease, circular RNAs, expression profiles

## Abstract

Circular RNAs (circRNAs), a novel kind of non-coding RNA, have received increasing attention for their involvement in pathogenesis of Alzheimer’s disease (AD); however, few studies have reported in the characterization and function of AD associated circRNAs. Here the expression profiles of circRNAs in 5- and 10-month-old SAMP8 mice were identified using circRNA microarray and found that 85 dysregulated circRNAs were observed in 10-month-old SAMP8 versus control mice and 231 circRNAs exhibited differential expression in 10-month-old SAMP8 versus 5-month-old SAMP8. One most significantly dysregulated circRNA, mmu_circRNA_017963, was select for Gene Oncology (GO) and pathway analysis. The results showed that mmu_circRNA_017963 was strongly related with autophagosome assembly, exocytosis, apoptotic process, transport and RNA splicing and highly associated with synaptic vesicle cycle, spliceosome, glycosaminoglycan and SNARE interactions in vesicular transport pathways. Collectively, this study was the first to describe circRNAs expression in different ages of SAMP8 and will contribute to the understanding of the regulatory roles of circRNAs in AD pathogenesis and provide a valuable resource for the diagnosis and therapy of AD.

## Introduction

Alzheimer’s disease (AD) is considered an age-related neurodegenerative disease and characterized neuropathologically by the presence of neuritic plaques and neurofibrillary tangles and clinically by a progressive impairment in cognitive function. In the United States alone, an approximate 476,000 people aged 65 and older develop AD in 2016 and this number is expected to grow to 13.8 million by mid-century [1]. Unfortunately, there is no effective therapeutic strategies preventing the progression of AD until now. Consequently, discovery of novel therapeutic targets, thereby providing novel strategies to treat AD is urgently needed.

Circular RNAs (circRNAs), a novel kind of RNA formed by special loop structure with neither a 5’cap nor a 3’ polyadenylated tail, are currently identified as noncoding RNAs (ncRNAs) which are highly represented in eukaryotic transcriptome [2]. CircRNAs are predominantly observed in the cytoplasm and can be sorted into exosomes [3]. Compared with microRNAs (miRNAs) and long noncoding RNAs (lncRNAs), circRNAs have a higher degree of stability and sequence conservation between different species [3,4]. There is growing evidence showing that circRNAs are not simply by-products of splicing errors or mis-spliced RNAs, but rather, they are the products of regulated back-spliced RNAs with distinct sets of trans-factors and/or cis-elements [5]. Recently, circRNAs have been shown to bind to microRNAs (miRNAs), acting as miRNA sponges to regulate gene expression at the transcriptional or post-transcriptional level [6,7]. Evidence is emerging that circRNAs have a close association with diseases and may play an important role in the pathogenesis and diagnosis of disease through miRNA sponges. For example, circ-ZNF609 can act as a sponge for miR-150-5p to modulate the expression of AKT3 in Hirschsprung disease [8]. In addition, hsa_circ_001569 acted as a positive regulator in cell proliferation and invasion of colorectal cancer (CRC) by sponging miR-145 [9]. Recently, circRNAs have been reported to contribute to AD pathogenesis. For instance, circRNA ciRS-7, containing more than 70 conserved miRNA target sites, can sponge miR-7 and thus depress the expression of ubiquitin carboxyl-terminal hydrolase L1 (UCHL1) gene, leading to degrade the protein levels of β-amyloid precursor protein (APP) and β-site APP cleaving enzyme 1 (BACE1), suggesting that ciRS-7 may represent a useful target in the development of therapeutic strategies for AD [6,10]. Additionally, Zhang, et al analyzed circRNA-associated-ceRNA network through deep RNA sequencing in AD model mouse and found that the circRNA-associated-ceRNA networks in AD mouse model were mainly involved in Aβ clearance (Hmgb2) and myelin function (Dio2) [11]. Up to now, only three studies have been reported about the roles of circRNAs in AD. Thus, identification of AD-associated circRNA profiles, and characterization and function of these circRNAs are necessary.

In the present study, we applied microarray technology to analyze the differential expression profiles of circRNAs in the hippocampus of three 10-month-old Senescence accelerated mice P8 (SAMP8) and senescence-accelerated mouse resistant R1(SAMR1) mice. We also identified differential circRNAs expression between 10-month-old and 5-month-old SAMP8 mice to assess the age-dependent dysregulation of circRNAs. We identified 85 differentially expressed circRNAs (45 upregulated and 40 downregulated) in 10-month-old SAMP8 versus age-matched SAMR1 mice and 231 differentially expressed circRNAs (110 upregulated and 121 downregulated) in 10-month-old SAMP8 versus 5-month-old SAMP8. From these differentially expressed circRNAs, six circRNAs were validated in SAMP8 mice by real-time qPCR . The miRNA binding sites of these confirmed circRNAs were uncovered, and the related mRNAs were predicted. Analyzing the differentially regulated circRNAs and their potential functions may contribute to the understanding of the regulatory roles of circRNAs in AD pathogenesis and provide a valuable resource for the diagnosis and therapy of AD in clinic.

## RESULTS

### Morris Water Maze (MWM) Test

**Figure 1:**
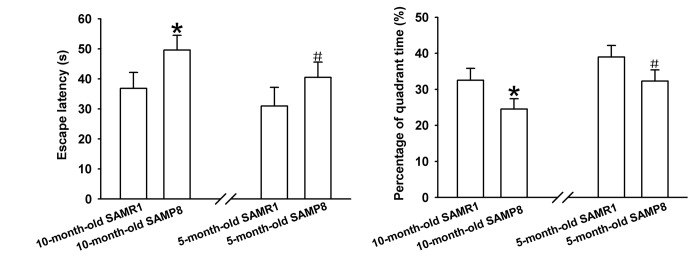
**Learning and memory ability evaluation by MWM test.** (**A**) Mean escape latency of four-day training. (**B**) The percentage of time spent in the target quadrant on day 5. *P<0.05 versus SAMP8 group.

The learning and memory abilities of SAMP8 mice were evaluated using MWM test. As shown in Figure 1A, the average escape latencies of four-day training in SAMP8 mice were significantly shorter than that in age-matched SAMR1 AD model mice respectively, implying that AD model mice have poor learning performances. Additionally, the percentage of time in the target quadrant of each mouse in the probe trail on day 5 was recorded to evaluate the memory ability after four-day training. As demonstrated in Figure 1B, the percentage of time spent in the target quadrant of SAMP8 AD mice markedly reduced compared to their control mice, indicating impaired memory abilities of AD model mice. Together, these results indicate that 5- and 10-month-old SAMP8 mice exhibited severe cognitive impairments.

### CircRNA expression profiles in hippocampal tissues of 10-month-old SAMP8 relative to age-matched SAMR1

**Figure 2:**
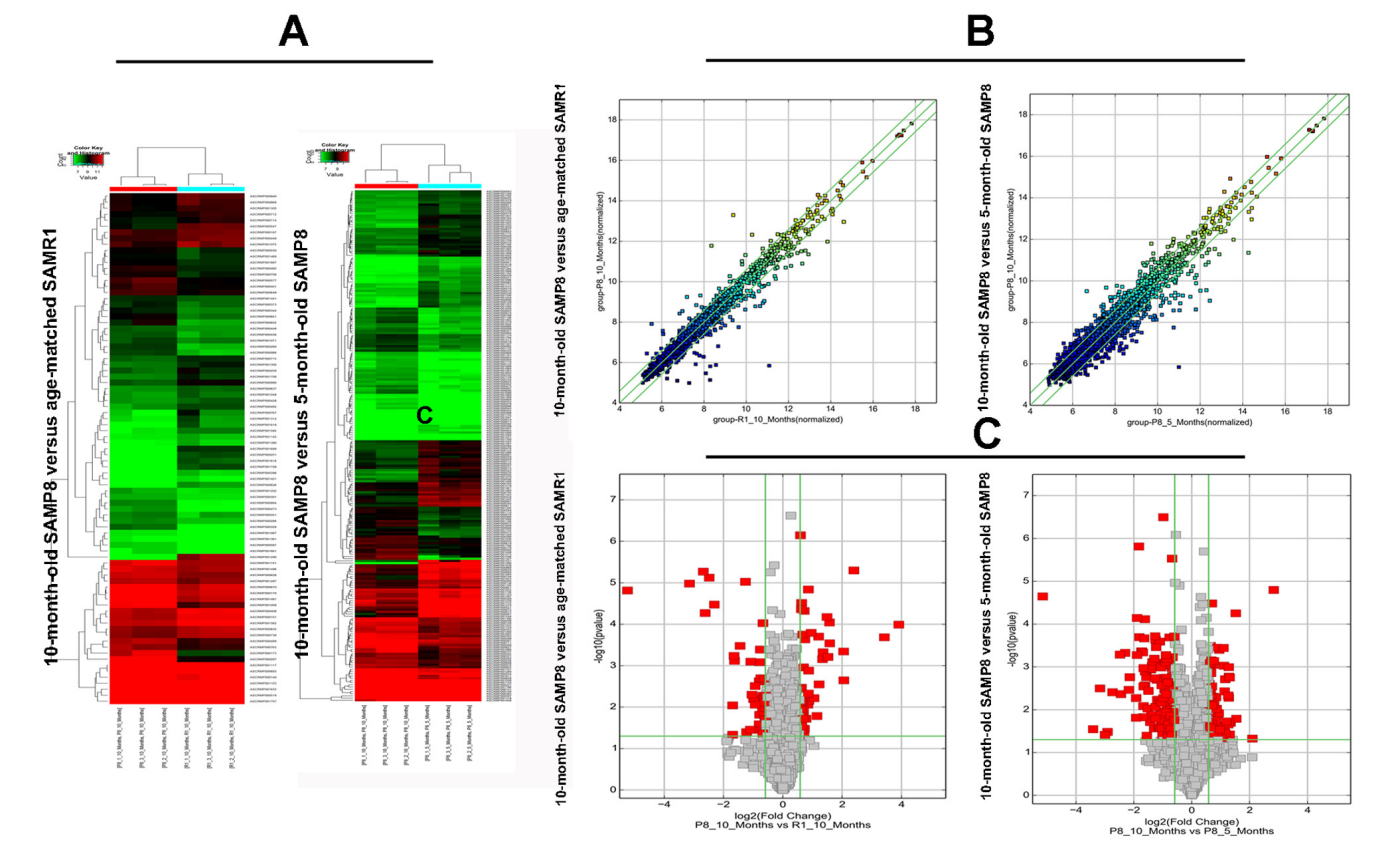
**The hierarchical cluster, scatter plot and volcano plot of differential expression of circRNAs in 10-month-old SAMP8 versus age-matched SAMR1 and 10-month-old SAMP8 versus 5-month-old SAMP8.** (**A**) Hierarchical cluster of differentially expressed circRNAs. “green” indicates low intensity, “black” indicates medium intensity and “red” indicates strong intensity. (**B**) Scatter plot of circRNA signal values. The values of X and Y axes represents the normalized signal values of the samples (log2 scaled) and the averaged normalized signal values of samples (log2 scaled) respectively. The green lines are fold change lines. The CircRNAs above the top green line and below the bottom green line demonstrates more than 1.5-fold change of circRNAs between the two compared samples. (**C**) Volcano plot of differential expression of circRNAs. The vertical lines correspond to 1.5-fold up and down, respectively. The horizontal line represents a *P*-value of 0.05, and the red point in the plot represents the differentially expressed circRNAs with statistical significance.

Significant difference was observed in circRNA expression patterns between hippocampal tissues of 10-month-old SAMP8 and age-matched SAMR1. 85 circRNAs were differentially expressed in hippocampal tissues of SAMP8 and SAMR1 (*P*<0.05). Among these aberrantly expressed circRNAs, 45 circRNAs were upregulated and 40 circRNAs were downregulated. CircRNA expression patterns of hippocampal tissues of SAMP8 and SAMR1 were classified by unsupervised hierarchical clustering as illustrated in Figure. 2A. A scatter plot depicting the variation in the expression of circRNAs between SAMP8 and SAMR1 hippocampal samples was showed in Figure.2B. Figure.2C showed a volcano plot that visualized the statistically significant difference of expressed circRNAs between two groups.

### CircRNA expression profiles in hippocampal tissues of 10-month-old SAMP8 relative to 5-month-old SAMP8

Expression levels of circRNAs were measured in 10-month-old SAMP8 mice relative to 5-month-old SAMP8 mice. 231 significantly dysregulated circRNAs were identified: 110 circRNAs were upregulated, while 121 circRNAs were down-regulated in 10-month-old SAMP8 samples (*P*<0.05). The hierarchical cluster, scatter plot and volcano plot of differential expression of circRNAs in 10-month-old SAMP8 and 5-month-old SAMP8 were shown in Figure 2A, B and C respectively.

### Validation of deregulated circRNA using real-time qPCR

**Figure 3:**
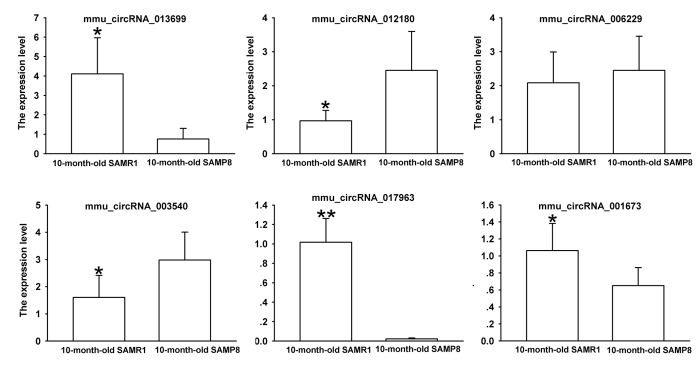
**The expression levels of candidate circRNAs for validation by real-time qPCR in 15 10-month-old SAMR1 and SAMP8 hippocampal tissues.** Statistically differences were calculated by *t*-test using SPSS 13.0 software. *P<0.05, **P<0.01 versus SAMP8 group.

**Figure 4:**
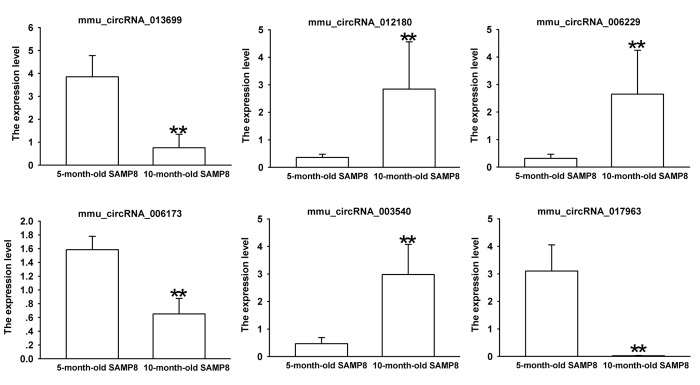
**The expression levels of candidate circRNAs for validation by real-time qPCR in 15 10-month-old SAMP8 and 5-month-old SAMP8 hippocampal tissues.** Statistically differences were calculated by *t*-test using SPSS 13.0 software. **P<0.01 versus 5-month-old SAMP8 group.

Since false positives can result from multi-comparisons, false discovery rate (FDR) method was performed to adjust *P* values. After FDR correction, 27 circRNAs in 10-month-old SAMP8 samples were significantly upregulated, while 18 circRNAs of SAMP8 samples were downregulated as compared to 10-month-old SAMR1 samples. 41 circRNAs were upregulated, while 78 circRNAs were downregulated in 10-month-old SAMP8 mice relative to 5-month-old SAMP8. In view of raw signal intensity (>500) and the fold change of expression (>2), six overlapped differentially expressed circRNAs in 10-month-old SAMP8 versus 10-month-old SAMR1 mice and 10-month-old SAMP8 versus 5-month-old SAMP8 mice were selected to validate their expression using real-time qPCR. The specificity of circRNA product amplified by divergent primers for these six circRNAs was tested by melting curve analysis. The results illustrated that the melting curve exhibited a single peak for each circRNA (Supplementary Figure 1), suggesting that there is no primer dimers or nonspecific amplified product in the amplified product and confirmed that the selected circRNAs could be amplified by real-time qPCR assay for validating the expression of selected circRNAs. The results of qPCR assay showed that five (mmu_circRNA_017963, mmu_circRNA_003540, mmu_circRNA_013699, mmu_circRNA_012180 and mmu_circRNA_006173) out of the six circRNAs significantly differed between the hippocampal tissues of 10-month-old SAMP8 and age-matched SAMR1 (*P*<0.05) (Figure 3). All of the selected circRNAs were significantly different in 10-month-old SAMP8 samples as compared to 5-month-old SAMP8 samples (Figure 4). It should be noted that the expression levels of mmu_circRNA_017963 in 10-month-old SAMP8 mice significantly decreased by factors of approximate 50 and 150 than those in age-matched SAMR1 and 5-month-old SAMP8 (Figure 3 and 4). Based on these results, mmu_circRNA_017963 was selected for further analysis.

### Annotation and prediction of mmu_circrna_017963 targeted miRNA-mRNA network

According to miRNA support vector regression (mirSVR) scores, five highest-ranking miRNA binding targets (“Top 5”) were identified for further analysis. The details of the molecular interactions between mmu_circRNA_017963 and its miRNA targets are described in Supplementary Figure 2. The predicted gene sequence of the best transcript of mmu_circRNA_017963 is Tbc1d30 and its genomic locus is on chromosome 10 based on the initial data of the microarray. The molecular interaction between Top-5 miRNA targets by mirSVR [12] and mmu_circRNA_017963 were predicted by its specific base pairing. We hypothesized that mmu_circRNA_017963 could regulate the activity of targeted miRNAs as miRNA sponges to modulate circRNA-miRNA-mRNA network, and that through miRWalk [13] and TargetScan [14], the interaction network could be predicted. In accordance with the above analysis tools, we predicted a total of 5 miRNAs and 313 mRNAs to interact with mmu_circRNA_017963.

**Figure 5:**
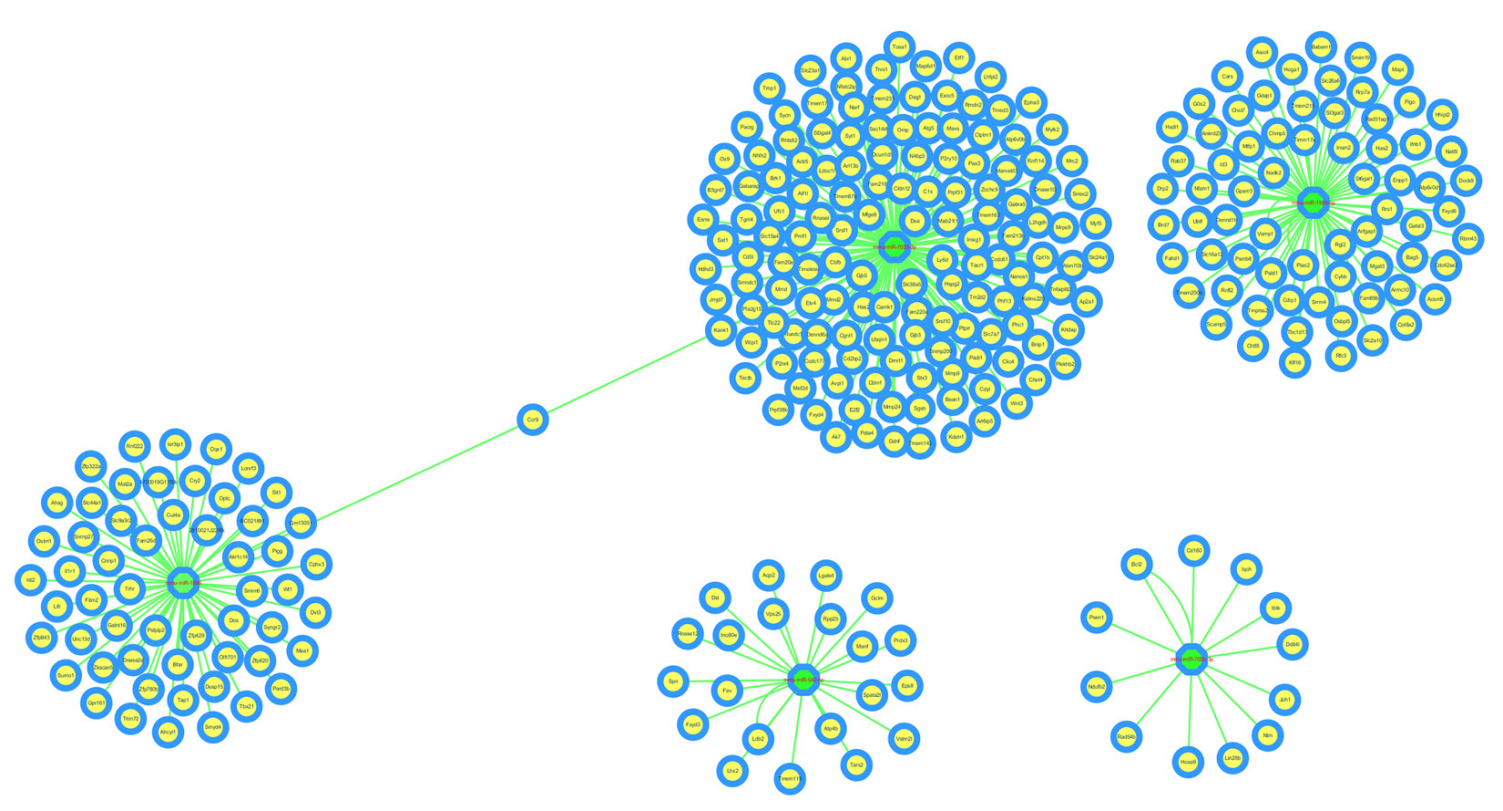
**The predicted mmu_circRNA_017963 targeted circRNA-miRNA-mRNA interaction network according to sequence-pairing prediction.** The miRNA-binding sites were predicted by mirSVR, and targeted miRNAs and mRNAs were predicted by miRWalk and TargetScan. Five miRNAs were observed with overlapping results.

**Figure 6:**
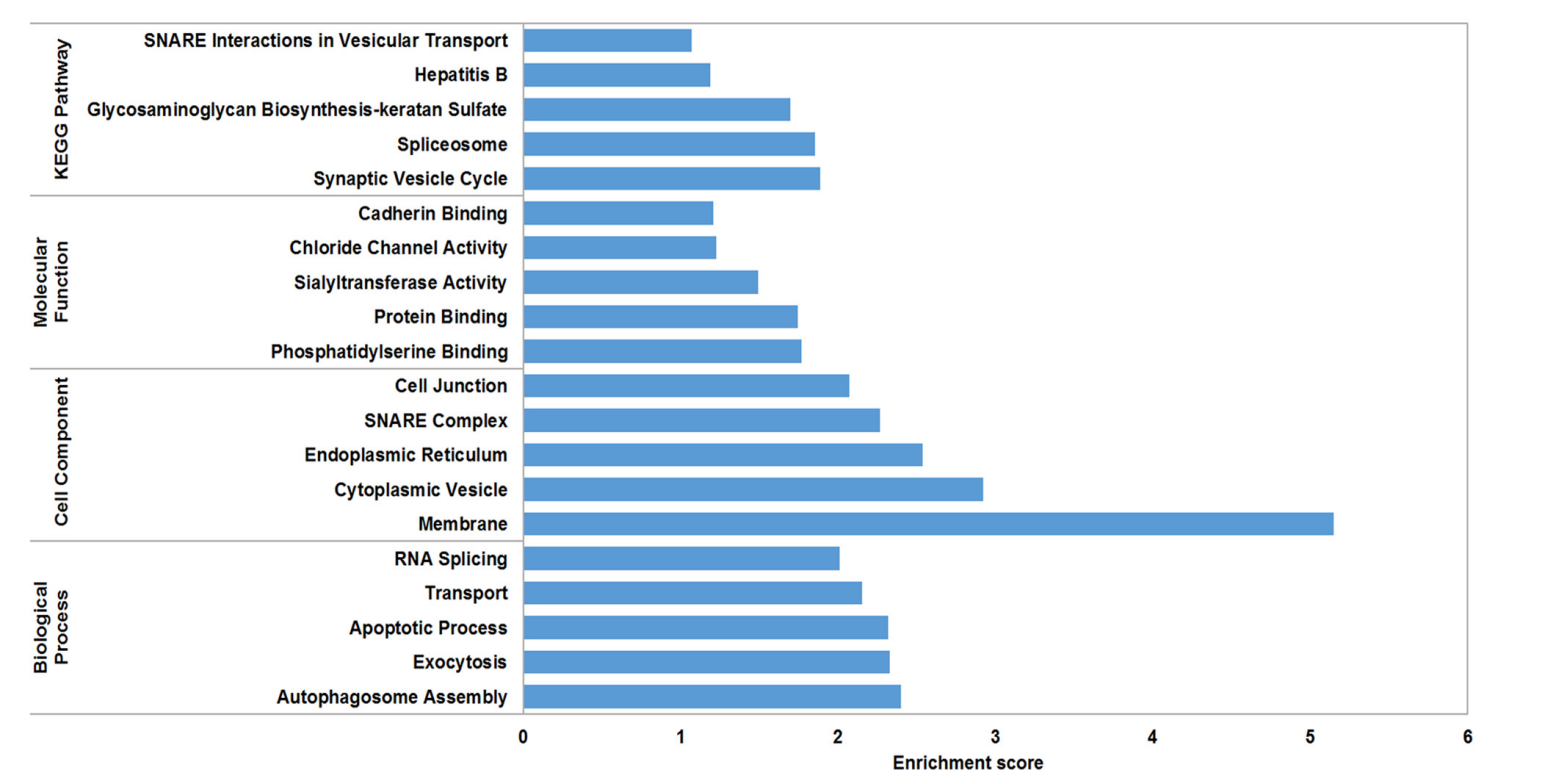
**GO analysis according to the mmu_circRNA_017963-miRNAs-mRNAs network.** The x- and y-axis represent the top 5 significantly enriched biological processes, cell component and molecular function and their scores (-log10 (*P* value)), respectively. The horizontal axis represents the significant level of GOs and KEGG pathways.

The circRNA-miRNA-mRNA interaction network of mmu_circRNA_017963 was analyzed using cytoscape software. As demonstrated in Figure 5, mmu_miR_7033-3p showed the largest interaction network, followed by mmu_miR_1955-5p, mmu_miR_7030-3p, mmu_miR_7033-3p and mmu_miR_542-5p. For further analyzing the functions of mmu_circRNA_017963, the KEGG and GO pathway analyses were performed according to the results predicted from miRWalk and TargetScan. GO analysis indicated that mmu_circRNA_017963 strongly related with autophagosome assembly (ontology: biological process, GO: 0000045), exocytosis (ontology: biological process, GO: 0006887), apoptotic process (ontology: biological process, GO: 0006915), transport (ontology: biological process, GO: 0006810) and RNA splicing (ontology: biological process, GO: 0008380) (Figure 6). Furthermore, the KEGG pathway enrichment analysis demonstrated that there are five signaling pathways, including synaptic vesicle cycle, spliceosome, glycosaminoglycan, Hepatitis B and N-ethylmaleimide-sensitive factor attachment protein receptor (SNARE) interactions in vesicular transport mediated pathways, were highly associated to the mRNAs predicted by the targeted miRNAs of mmu_circRNA_017963(Figure 6).

## DISCUSSION

AD is a chronic neurodegenerative disease characterized by a decline in memory, language, motivation and problem-solving [15]. Although studies have made progress in AD pathogenesis, the pathogenic mechanism of AD is still unclear. For the past few years, many studies have concentrated on the epigenetic regulation of AD pathogenesis, such as the contributions of DNA methylation and lncRNAs to AD. Recently, the occurrence of circRNAs in AD attracts increasing attention [10,11]. However, very little is known about the roles of circRNAs in AD. Consequently, systematic dissection of circRNA profiling in AD may benefit to understanding the roles of dysregulated circRNAs in AD pathogenesis.

SAMP8 shows significant age-related deteriorations in memory and learning ability in accordance with early onset and rapid advancement of senescence. Neuropathological changes, including abnormal Aβ accumulation and impaired Aβ clearance, hyperphosphorylation of tau protein, and increased oxidative stress and gliosis, were observed in various brain regions [16]. Thus, SAMP8 has been considered as an appropriate AD model to understand the pathomechanisms of AD, especially sporadic AD (SAD) [17,18]. Our MWM test showed that 5-month-old SAMP8 and 10-month-old SAMP8 exhibited an impaired cognitive function when compared to their age-matched strain SAMR1 (Figure 1), which confirmed the results of previous studies [16,18]. On the contrary, the transgenic AD mouse models, such as 2xTg-AD (APP/PS1) and 3xTg-AD (APP/PS1/Tau), may simply represent uncommon familial AD (FAD) [19]. Since SAD constitutes more than 95% of all AD cases, thus in the present study, 5 and 10-month-old SAMP8 mice were performed to identify the expression profiles of circRNAs using high-throughput circRNA microarray. The results showed that there were 85 differentially expressed circRNAs (45 upregulated and 40 downregulated) in the hippocampus tissues of 10-month-old SAMP8 and age-matched SAMR1(*P*<0.05). Since AD is an age-related neurodegenerative disease, in order to assess the age-dependent dysregulation of circRNAs, differential circRNA expression between 10-month-old SAMP8 and 5-month-old SAMP8 were measured. The results demonstrated that 231 circRNAs were significantly dysregulated (110 upregulated and 121 downregulated) in 10-month-old SAMP8 relative to 5-month-old SAMP8. Six overlapped differentially expressed circRNAs in 10-month-old SAMP8 versus 10-month-old SAMR1 and 10-month-old SAMP8 versus 5-month-old SAMP8 were then chosen for validation. The results showed that mmu_circRNA_017963, mmu_circRNA_003540, mmu_circRNA_013699, mmu_circRNA_012180 and mmu_circRNA_006173 in the hippocampus of 10-month-old SAMP8 were significantly different compared to that of age-matched SAMR1 (Figure 3). Additionally, all of the selected circRNAs were validated to be differentially expressed in 10-month-old SAMP8 versus 5-month-old SAMP8 samples (Figure 4). It should be noted that mmu_circRNA_017963, mmu_circRNA_003540, mmu_circRNA_013699, mmu_circRNA_012180 and mmu_circRNA_006173 were significantly dysregulated in 10-month-old SAMP8 versus 10-month-old SAMR1 mice and 10-month-old SAMP8 versus 5-month-old SAMP8 mice, implying that these significantly dysregulated circRNAs in an age-dependent manner might facilitate the AD progression. However, the dysregulatory impacts of normal aging could not be excluded. In future studies, 5-month-old control mice should be involved to pinpoint the age-dependent dysregulatory influences in SAMP8 AD mice. Collectively, the above results suggest that there are plentiful aberrantly expressed circRNAs in AD and these differentially expressed circRNAs may participate in AD pathogenesis.

CircRNAs have more miRNA binding sites and may have more impacts on sequestering miRNAs compared to linear miRNA sponges, and thus by acting as miRNA sponges, circRNAs may regulate linear RNA transcription and protein production [20,21]. Therefore, through targeted miRNA-mediated effects on gene expression, circRNAs may play a vital role in the pathogenesis of AD. In the study, it was found that mmu_circRNA_017963 might potentially interact with five miRNAs including mmu_miR_1896, mmu_miR_1955-5p, mmu_miR_7030-3p, mmu_miR_7033-3p and mmu_miR_542-5p. Since few studies have focused on the relationship between these sponged miRNAs and AD up to now, for further identifying the function of mmu_circRNA_017963, cytoscape was used to analyze the circRNA-miRNA-mRNA interaction network of mmu_circRNA_017963, the result of which indicated that mmu_miR_7033-3p showed the largest interaction network (Figure 5). Moreover, GO and pathway analysis were performed on the miRNA-targeted genes associated with the significant circRNAs to predict the potential biological, cellular, cell component and molecular functions of these circRNAs. GO analysis revealed that mmu_circRNA_017963 has a high probability of participating in the biological processes of autophagosome assembly, exocytosis, apoptotic process, transport and RNA splicing (Figure 6). All of these biological processes are reported to have a vital role in the development of AD [22-26]. KEGG pathway analysis indicated that mmu_circRNA_017963 are involved in the pathways of synaptic vesicle cycle, spliceosome, glycosaminoglycan, Hepatitis B and SNARE interactions in vesicular transport. Among these signaling pathways, the pathways of synaptic vesicle cycle, spliceosome, glycosaminoglycan and SNARE interactions in vesicular transport have been proven to have association with AD pathogenesis. For example, it has been proven that alternative splicing of genes may result in incorrect APP processing and increased β-amyloid production [27], indicating that spliceosome pathway may contribute to AD pathogenesis. Studies demonstrated that synaptic vesicle-related gene expression reduced in AD brain, which may result in synapses loss and cognitive decline in AD [28]. There are reports showing that sulphated glycosaminoglycans can stimulate tau phosphorylation by a number of protein kinases, and lead to the formation of the neurofibrillary lesions of AD [29]. Studies have shown that SNARE is crucial for neuroexocytosis, aberration in SNARE complex formation elicits erratic neuronal activity, which could cause cognitive impairments [30]. It has been reported that Aβ oligomers can directly inhibit SNARE complex formation in AD cell and mouse models, which may lead to cognitive deficits in AD [31]. Accordingly, in light of these findings, It can be hypothesized that mmu_circRNA_017963 may be involved in AD-associated signaling pathways. Nevertheless, the potential functions of mmu_circRNA_017963 in AD pathogenesis remains unknown. Therefore, further investigation of mmu_circRNA_017963 is needed.

Some limitations have to be interpreted in our results. Firstly, when evaluating the expression of circRNAs, false negative is inevitable since the minimum detection thresholds is relatively low due to the low level of circRNAs. Secondly, the expressions of the selected circRNAs should be validated in human samples and the circRNA-targeted miRNAs and mRNAs should be experimentally identified in further studies. Thirdly, since circRNAs are more stable than linear RNAs, circRNA level in peripheral blood samples should be measured and the diagnostic value of circRNA should be evaluated in further study.

This study was the first to describe the expression of circRNAs in different ages of SAMP8 mice. The results illustrated that circRNAs were differentially expressed in SAMP8 AD model as compared to control mice. Additionally, circRNAs also showed differential expressions in SAMP8 mice in an age-dependent manner. This study will make contribution to the understanding of the regulatory roles of circRNAs in AD pathogenesis and provide a valuable resource for the diagnosis and therapy of AD in clinic.

## METHODS

### Animals

5- and 10-month-old SAMP8 and their age-matched SAMR1 (control strain of SAMP8) mice were purchased from Tianjin University of Traditional Chinese Medicine (Tianjin, China). All the mice were pathogen- and virus-free. They were housed under the condition of 12-h light/dark cycle at 25 °C and 50 ± 10% relative humidity. Animal care and experimental procedures were implemented according to the document “Guidance Suggestions for Caring for Laboratory Animals” produced by the Ministry of Science and Technology of China in 2006.

### MWM Test

The spatial learning and memory of SAMP8 AD model mice were evaluated through the MWM test as previously described in our previous study [32]. Briefly, the mice were subjected to four consecutive daily training trials to test the capacity of spatial learning. In each trial, the mice were randomly introduced into the water at one of four start locations and trained to locate the hidden platform with a maximum trial time of 60 seconds and an interval of approximately 30 seconds. On day 5, the memory abilities of mice were tested. The mice were placed to a water pool without the hidden platform and started at the start location in the quadrant opposite to the quadrant in which the former platform was placed. The percentage of time spent in the former platform quadrant was recorded.

### Preparation of tissues

After MWM test, mice were ethically sacrificed and their hippocampal tissues were excised and stored at −80 °C prior to analysis. Animal care and experimental procedures were implemented according to the document “Guidance Suggestions for Caring for Laboratory Animals” produced by the Ministry of Science and Technology of China in 2006.

### RNA extraction and reverse transcription

Total RNA was extracted from each hippocampal tissue in TRIzol reagent (Invitrogen). For reverse transcription, 2μg total RNA, 4μL 5×RT Buffer, 1μL RT Enzyme Mix, 1μL Primer Mix, and RNase free water were contained in the reaction system according to the instruction of ReverTra Ace qPCR RT Kit (Toyobo).

### CircRNA microarray

Three hippocampal tissues of SAMP8 and SAMR1 were used for microarray assay to determine differentially expressed circRNAs using the circRNAs chip (Arraystar mouse circRNAs chip, AraryStar) containing 14,236 probes specific for mouse circular RNAs splicing sites. The microarray hybridization including purifying RNA, transcribing into fluorescent cRNA was performed based on the manufacturer’s standard protocols and then hybridizing onto mouse circRNA arrays. After the hybridized slides were washed and fixed, the slides were scanned using Agilent Scanner G2505C, followed by the data collection by Agilent Feature Extraction software.

### Microarray analysis

The raw data were normalized using the Kangcheng homemade R software package (Kangcheng Bio-tech, Shanghai, China) and the statistical significance of differentially regulated circRNAs between SAMP8 and SAMR1 mice was identified according to fold change cutoff and *P*-value (*P*<0.05) or through the Volcano Plot filtering. After prediction of miRNA targets of circRNAs and the circRNA/miRNA interaction based on miRWalk [33] and TargetScan [14], the mirSVR algorithm was used to score and rank the efficiency of the predicted miRNA targets. 5 miRNAs with the highest mirSVR score were identified for the establishment of ‘‘Top5’’ circRNA-miRNA network [34].

### Real-time qPCR

Considering the fold change of expression tested in the microarray analysis and the predicted target miRNAs related with progression of AD in previous research, 6 differentially expressed circRNAs (mmu_circRNA_017963, mmu_circRNA_003540, mmu_circRNA_013699, mmu_circRNA_012180, mmu_circRNA_006229 and mmu_circRNA_006173) were selected for further investigation. The selected circRNAs were validated using real-time qPCR in triplicate in 15 samples of SAMP8 mice. Divergent primers of the selected circRNAs were designed and optimized according to the sequences of the circRNAs obtained from the database “circBase” (http://circbase.mdc-berlin.de). β-action (ACTB), a housekeeping gene, was used as a control. The primer sequences were as follows: 5’- TGACTGACGGCTCTGTTCTG-3’ (sense) and 5’- CAATTGTTACCTGCCCCATC-3’ (anti-sense) for mmu_circRNA_003540, 5’- AAAGCTCACCCAGGACCAGTT-3’ (sense) and 5’- TATGTTTCCTCGCTCAGTGCC-3’ (anti-sense) for mmu_circRNA_013699, 5’- TGCGAGACAGCAGACGAAT-3’ (sense) and 5’- TTTGTATCAACTCCTGCATGAG-3’ (anti-sense) for mmu_circRNA_017963, 5’- ACTGCACTCAAGGTCCAACA-3’ (sense) and 5’- CCATATTCTTCATAGCTGAGCA-3’ (anti-sense) for mmu_circRNA_012180, 5’- GCATTTTACTGAAGGAGCCG-3’ (sense) and 5’- GAGGACCATGCTATTCTGGAAG-3’ (anti-sense) for mmu_circRNA_006229, 5’- TCTACCAGGCTCTCCTCCCA-3’ (sense) and 5’- TCCAGGCACGTGCTCTGAG-3’ (anti-sense) for mmu_circRNA_006173. The appearance of a single peak in the melting curve of each sample demonstrated the specificity of the qPCR results. The relative gene expression was calculated by 2 ^–ΔΔCp^ method, where ΔΔCp = ΔCp ^treatment^ − ΔCp ^control^ and ΔCp = Cp ^target gene^ − Cp ^𝐴CT𝐵 gene^ [35,36].

### Annotation and function prediction

According to the qPCR results, mmu_circRNA_017963 was selected for annotation and function prediction based on circRNA-miRNA-gene network in the light of the analysis of miRWalk (http://zmf.umm.uni-heidelberg.de/apps/zmf/mirwalk2/) and TargetScan (http://www.targetscan.org/). In addition, cytoscape (http://www.cytoscape.org/) was utilized to establish a circRNA-miRNA-mRNA interaction network of mmu_circRNA_017963. Functional annotation of genes in the networks were performed using KEGG and GO pathway analysis.

### Statistical analysis

Analysis of variance was performed with SPSS software for windows 13.0. The significance of real-time qPCR validation between the hippocampal tissues of SAMP8 and SAMR1 was tested by the Student *t* test, and *P* <0.05 was considered statistically significant.

## Supplementary Material

Supplementary File
