# Supplementary material for: Comprehensive analysis of differentially expressed profiles of Alzheimer’s disease associated circular RNAs in an Alzheimer’s disease mouse model
Source: Aging (Albany NY). 2018 Feb 15;10(2):253–65. doi: 10.18632/aging.101387 (PMC5842852; doi:10.18632/aging.101387)
Supplement: Supplementary File [file aging-10-101387-s001.pdf]

SUPPLEMENTARY MATERIAL

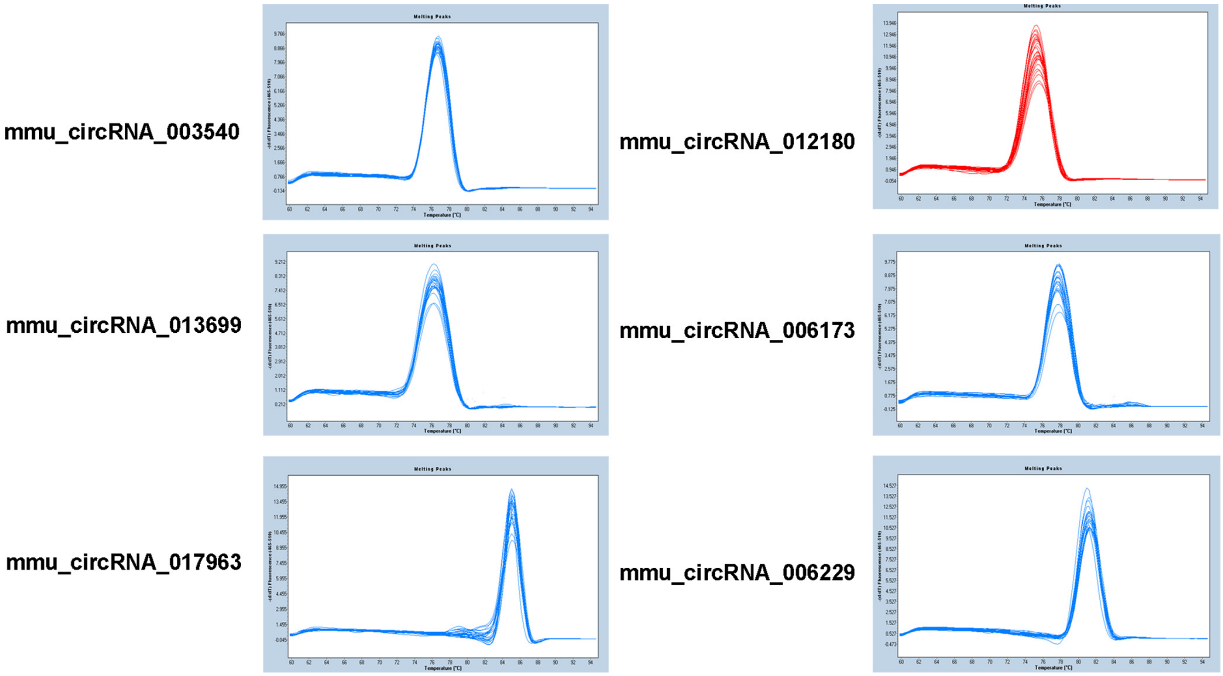

Supplementary Figure 1. The melting curve of circRNAs.

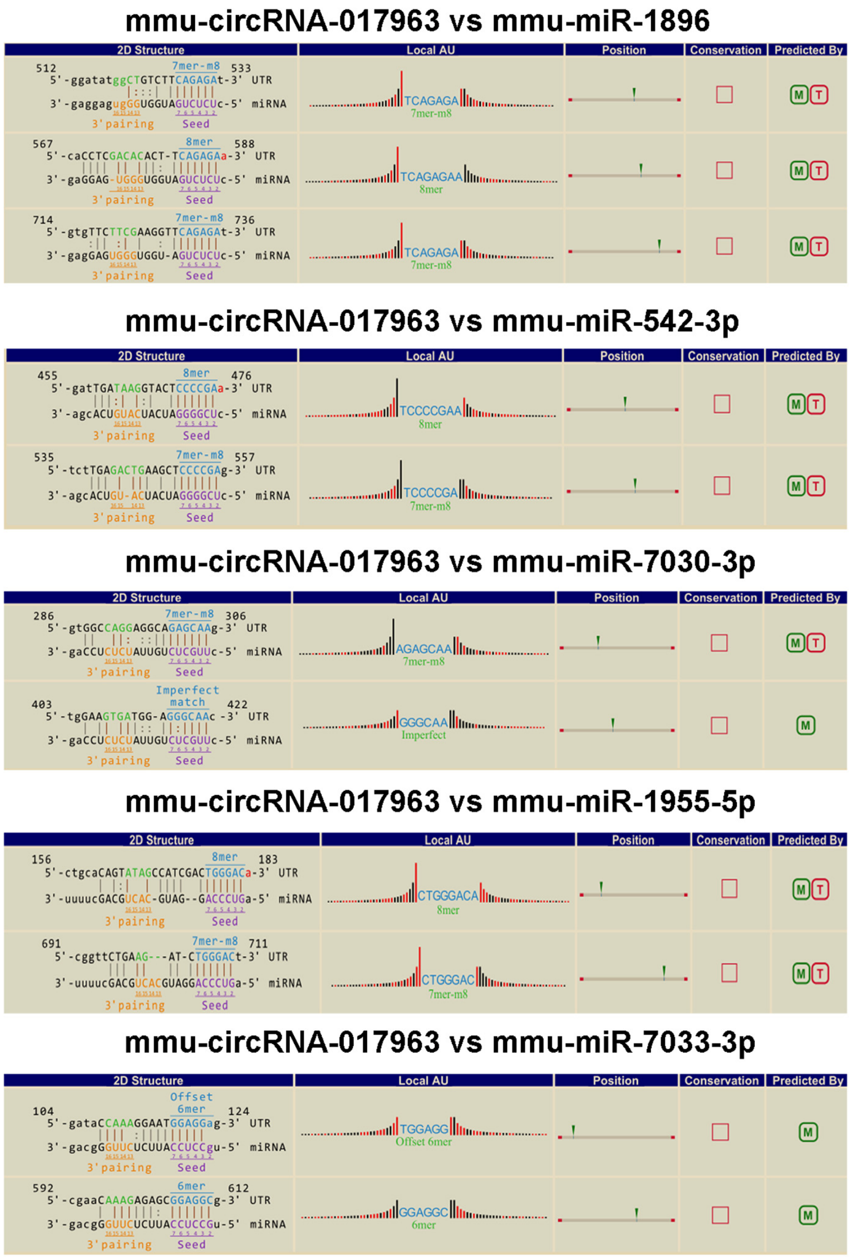

Supplementary Figure 2. Five detailed annotation for the circRNA/miRNA interaction (mmu_circRNA_017963 and its Top-5 predicted miRNA targets).
